# Weekly influenza-like-Illness rates were significantly lower in areas where schools were not in session in the United States during the 2009 H1N1 pandemic.

**DOI:** 10.1371/currents.RRN1234

**Published:** 2011-04-27

**Authors:** Olivia Briffault

**Affiliations:** Hunter College High School, New York, United States

## Abstract

This study investigated the relationship between school session status and H1N1 influenza prevalence. Weekly means of Influenza-like-Illness (ILI) rates over the period May 1 to October 31,2009 were compared between areas where schools were and were not in session in the United States. Rates were substantially and significantly higher in areas where schools were in session. This result held separately in spring and fall and was robust to various controls.

Background:

One potential non-pharmaceutical control measure against the spread of influenza is social distancing, through measures such as school closure [1] .  The rationale for school closure as a control measure is the assumption that flu spreads less rapidly when children are not in school [2] .  There is, however, limited evidence in support of this assumption.   Four prior studies, Cowling et al., Cauchemez et al., Wu et al and Chao et al. examined the question of whether influenza spreads less rapidly when children are not in school [3] ^[4]^ .  Cowling et al.  examined the consequences of Hong Kong’s decision to close local schools at the height of the 2008 winter seasonal influenza epidemic [3] .  They found that school closures were associated with lower influenza rates [3] .   Cauchemez et al. examined how the regional pattern of school holidays in France related to the rates of influenza-like-illness [4] . They found that during holidays, influenza-like-illness rates were lower [4]. Wu et al examined the H1N1 Pandemic of 2009 in Hong Kong [5]. They examined how pandemic spread was influenced by school closures associated with summer vacation in Hong Kong [5]. Chao et al examined how pandemic influenza transmission was influenced by the reopening of schools after summer vacation during the fall 2009 H1N1 pandemic [6].  Chao et al found that transmission was significantly higher after schools reopened and that school reopening may have brought on a second wave of the pandemic [6].

This study follows Cauchemez et al. and Chao et al., by using variability in school calendars across the United States to examine the relationship between school session status (whether children are in school or not) and influenza rates. It replicates the results of Chao et al. for fall 2009, and extends the analysis by also examining, like Wu et al., school closures before summer vacation. The beginning of summer vacation and the end of summer vacation each vary by about five weeks across the country.  This variability predates the H1N1 virus, but offers an opportunity to examine how school attendance affected its spread.

Methods:

The sample period for this study was May 1, 2009 and October 31, 2009, a period during which the main variant of influenza circulating was H1N1 [5].   Influenza-Like Illness (ILI) rates, the percentage of emergency department or outpatient visits associated with ILI, were obtained from local public health department websites for 21 areas across the country that report ILI data publicly and also report to the International Society for Disease Surveillance database. The areas are: Alabama, Arizona, Boston, Colorado, Connecticut, Florida, Georgia, Indiana, Maine, Minnesota, Missouri, North Carolina, North Dakota, New York City, Pennsylvania, Rhode Island, Texas, Utah, Virginia, Washington, and Wisconsin.  Data for ILI rates was available in chart form on these websites and rates were read off the graphs and confirmed by a second reader.  School calendar data for corresponding areas was obtained from school websites. In areas that included multiple school districts with different closing dates, this study chose the school closure date of the largest school district in the most populous county. Table 1 provides the websites used to obtain the ILI rates and the school closure dates. Note that school calendars are generally removed from the websites at the end of each school year. All information here was obtained October/November 2009 when 08-09 and 09-10 calendars were still available.

**Figure:**
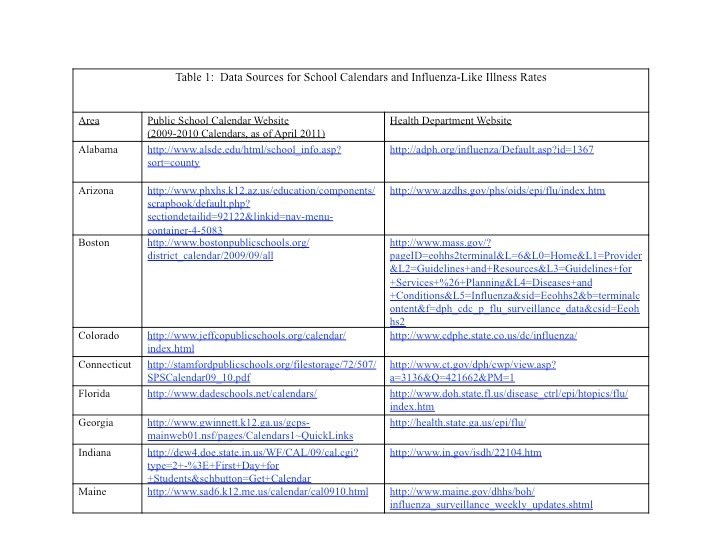

**Figure:**
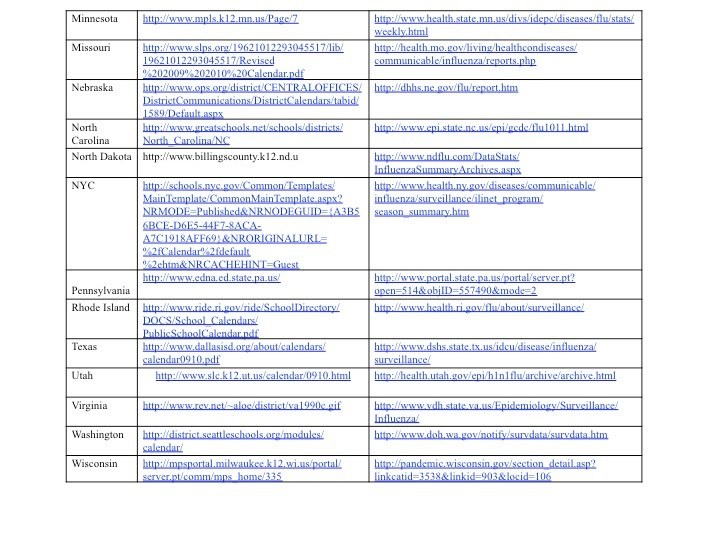

Weekly ILI rates where schools were in session were compared to rates where schools were not in session using a Mann-Whitney-Wilcoxon test [6].  The analysis was conducted initially for the full May-October period, and then separately for those weeks in spring and fall where school session status varied.  In some areas, ILI data were reported from emergency rooms while others included outpatient visits.  To adjust for such area differences, the analyses were repeated using deviations from each area’s mean ILI rate.  There may also be weekly variability in average ILI rates, associated, for example, with seasonality.  To adjust for this possibility, the analyses were repeated using deviations from the weekly mean ILI rate.   Finally, the analyses for fall were repeated omitting areas with high ILI rates (defined as rates above 5% for more than three weeks) during the preceding spring to adjust for the potential that populations in these areas may have acquired immunity to the virus.  

Results:

Figure 1 shows the number of areas with schools open and with schools closed each week. Note that during week 22 and week 34 the number of areas with schools open and the number of areas with schools closed was about equal.

**Figure:**
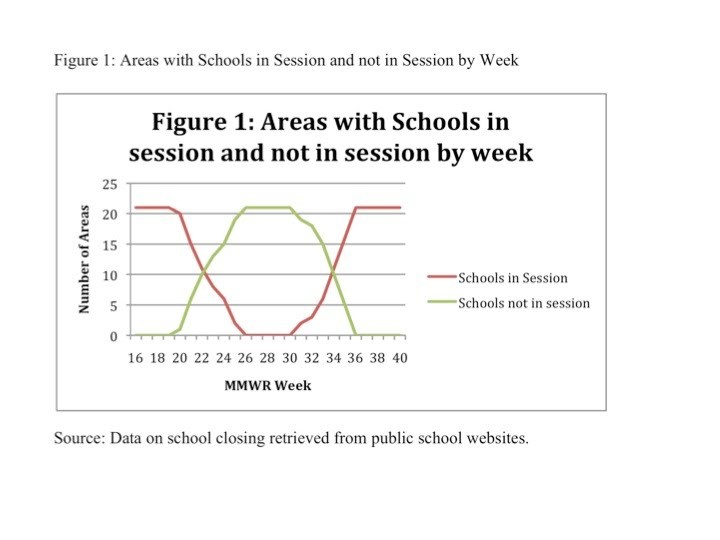

Figure 2 plots the average rate of ILI by week for areas where schools were in session and areas where schools were not in session and the 99% confidence interval for both series. 

**Figure:**
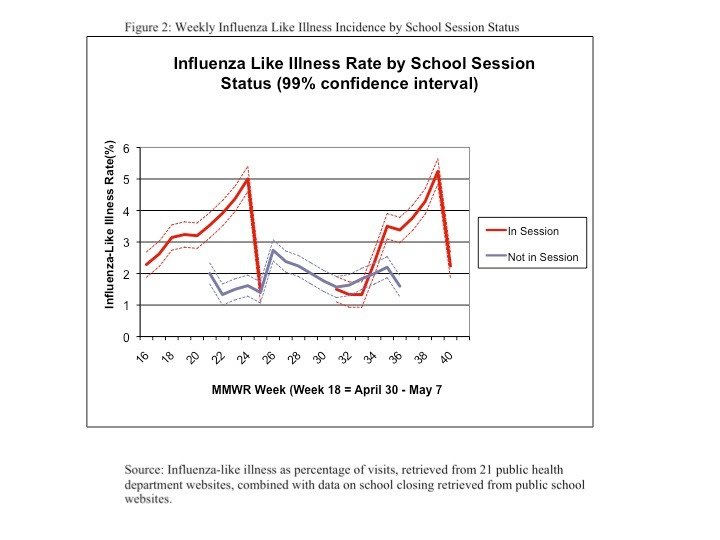

  Between May 15 and June 18, 2009  (weeks 20-25) and between July 30 and August 27, 2009 (weeks 30-35) some areas had schools in session and other areas had schools not in session.  Over the entire sample, the average ILI rate was 2.8%.  In areas where schools were in session during these periods, the average ILI rate was about 3.5%, while in areas where schools were not in session, the average ILI rate was 1.5 percentage points lower, about 2% (p<0.01).   ILI rates were higher in areas where schools were in session in almost all weeks. The exceptions were during weeks 25 and weeks 31-33. During these weeks there were very few (2, 2, 3 and 6 respectively) areas where schools were in session.    

Figure 3 presents the differences in mean weekly ILI rates between areas where schools were in session and those where they were not in session for the full period, and separately for both spring and fall. 

**Figure:**
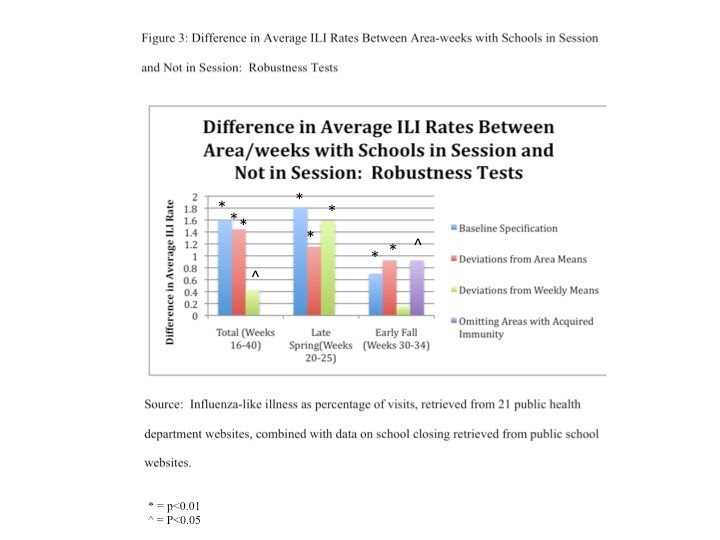

Over the full period, the difference in rates was 1.6 percentage points (p<0.01).  In the spring, the difference was 1.8 percentage points  (p<0.01), while in the fall, the difference was 0.7 percentage points (p<0.01).  When the analysis was repeated using deviations from area means, the levels and statistical significance remained comparable. When the analysis was repeated using deviations from weekly means, the magnitude for the results for the full sample was about a quarter as large and the significance level fell to p<0.04.  The results for the spring were comparable to the original results in magnitude and significance, but, after this adjustment, the results for fall were no longer statistically significant. Omitting areas with high ILI rates in spring had little effect on the fall rates, which remained statistically significantly higher at P<0.03 and of similar magnitude.  

Conclusion:

These findings suggest that influenza rates are about 1.7 times higher when children are in school than when they are not. The results were stronger for school closing in the spring than school reopening in the fall.  The results for spring may have been strengthened by the greater geographic variability of the initial influenza outbreak. The lack of significance of the fall results after controlling for calendar week suggest that total influenza rates across the country were rising at the same time that schools were opening.  While the results suggest an association between school session status and influenza spread, they do not provide an explanation for this pattern. The results are consistent with the view that in the event of a severe flu outbreak, ending school sessions early or delaying student return to school could help control virus spread, at least briefly.  The study has several limitations.  There may have been measurement error in the calculation of ILI rates, as these were read from graphs published by state health departments.  Second, in some states the opening and closing dates vary across school districts and this study assigned only one opening and closing date (that of the largest school district) per state   Finally, this study examined the effect of school session status, not the effect of closing schools because of an outbreak. It is possible that the results would be different in the latter situation. 

 Acknowledgements: 

I would like to acknowledge the invaluable assistance I've received from my mentor, Dr. Sherry Glied at the Department of Health Policy and Management of the Mailman School of Public Health at Columbia University in writing and revising this article.   

Funding:

There was no outside funding for this research. 

Competing interests: The author has declared that no competing interests exist.

## References

[ref-2886828875] Aledort JE, Lurie N, Wasserman J, Bozzette SA. Non-pharmaceutical public health interventions for pandemic influenza: an evaluation of the evidence base. BMC Public Health 2007;7:208. 10.1186/1471-2458-7-208 PMC2040158 17697389

[ref-863348492] Morse SS, Garwin RL, Olsiewski PJ. Public health. Next flu pandemic: what to do until the vaccine arrives? Science 2006;314(5801):929. 10.1126/science.1135823 17095681

[ref-2326517117] Cowling BJ, Lau EH, Lam CL, et al. Effects of school closures, 2008 winter influenza season, Hong Kong. Emerg Infect Dis 2008;14(10):1660-2. 10.3201/eid1410.080646 PMC2609897 18826841

[ref-1137246769] Cauchemez S, Valleron AJ, Boelle PY, Flahault A, Ferguson NM. Estimating the impact of school closure on influenza transmission from Sentinel data. Nature 2008;452(7188):750-4. 10.1038/nature06732 18401408

[ref-1879708675] Wu JT, Cowling BJ, Lau EH, et al. School closure and mitigation of pandemic (H1N1) 2009, Hong Kong. Emerg Infect Dis 2010;16(3):538-41. 10.3201/eid1603.091216 PMC3206396 20202441

[ref-2815500790] Chao DL, Halloran ME, Longini IM, Jr. School opening dates predict pandemic influenza A(H1N1) outbreaks in the United States. J Infect Dis 2010;202(6):877-80. 10.1086/655810 PMC2939723 20704486

[ref-3024469377] United States Centers for Disease Control and Prevention. The Influenza (Flu) Viruses. Available at: <http://www.cdc.gov/flu/about/viruses/index.htm>. . Accessed December 11 2009.

[ref-601615231] Boersma P. Wilcoxon Test. Available at: <http://www.fon.hum.uva.nl/Welcome.html>. . Accessed November 30 2009.

